# Heart failure and microvascular dysfunction: an in-depth review of mechanisms, diagnostic strategies, and innovative therapies

**DOI:** 10.1097/MS9.0000000000002971

**Published:** 2024-12-24

**Authors:** Ajeet Singh, Saad Ashraf, Hamza Irfan, Fnu Venjhraj, Amogh Verma, Ayesha Shaukat, Muhammad Daoud Tariq, Hafiz Muhammad Hamza

**Affiliations:** a Department of Internal Medicine, Dow University of Health Sciences, Karachi, Pakistan; b Department of Medicine, Dow University of Health Sciences, Karachi, Pakistan; c Department of Ophthalmology, Shaikh Khalifa Bin Zayed Al Nahyan Medical and Dental College, Lahore, Pakistan; d Shaheed Mohtarma Benazir Bhutto Medical College Lyari, Karachi, Pakistan; e SR Sanjeevani Hospital, Kalyanpur, Siraha, Nepal; f Department of Internal Medicine, Foundation University Medical College, Islamabad, Pakistan; g Foundation University School of Health Sciences, Islamabad, Pakistan

**Keywords:** coronary flow reserve (CFR), coronary microvascular dysfunction (CMD), heart failure with preserved ejection fraction (hFpEF), heart failure with reduced ejection fraction (hFrEF), microvascular dysfunction

## Abstract

Microvascular dysfunction (MVD) is increasingly recognized as a critical contributor to the pathogenesis of heart failure (HF), particularly in heart failure with preserved ejection fraction (HFpEF) and heart failure with reduced ejection fraction (HFrEF). Coronary microvascular dysfunction (CMD) significantly impacts HFpEF by reducing coronary flow reserve and myocardial perfusion reserve, leading to adverse outcomes such as myocardial ischemia, diastolic dysfunction, and increased risk of major cardiovascular events, including atrial fibrillation. In HFrEF, microvascular impairment is linked to heightened oxidative stress, reduced nitric oxide production, and activation of the renin-angiotensin-aldosterone system, further driving disease progression and contributing to poor prognosis. Advancements in diagnostic techniques, such as positron emission tomography, cardiac magnetic resonance imaging, and biomarker analysis, improve our ability to assess CMD in heart failure patients, enabling earlier diagnosis and risk stratification. Emerging therapies, including sodium-glucose cotransporter-2 inhibitors, angiotensin receptor-neprilysin inhibitors, and endothelial-targeted interventions, enhance microvascular function and improve patient outcomes. The role of personalized medicine is becoming increasingly important, as individualized therapeutic approaches tailored to patient-specific microvascular abnormalities are essential for optimizing treatment effectiveness. This review underscores the pivotal role of MVD in HF. It highlights the urgent need for innovative therapeutic strategies and diagnostic tools to address this complex condition and improve clinical outcomes for HF patients.

## Introduction

**Highlights**

- Microvascular dysfunction (MVD) is a significant driver in both heart failure (HF) with preserved ejection fraction and reduced ejection fraction. It impairs coronary microcirculation, leading to inadequate myocardial perfusion and exacerbating HF symptoms and progression.
- Emerging imaging techniques such as positron emission tomography and cardiac magnetic resonance imaging are crucial for assessing MVD, enabling earlier diagnosis and risk stratification. Combining these with biomarker analysis offers a comprehensive understanding of microvascular health.
- Emerging therapies such as sodium-glucose cotransporter-2 inhibitors and angiotensin receptor-neprilysin inhibitors have shown promise in improving microvascular function and HF outcomes.
- There is a significant gap in understanding the mechanisms underlying MVD and its impact on HF progression. Enhancing knowledge of MVD mechanisms and implementing personalized treatment approaches can improve outcomes and quality of life for HF patients with microvascular abnormalities.
- Future research should focus on bridging gaps in understanding, addressing challenges in translating findings into clinical practice, and exploring new diagnostic and therapeutic strategies.

According to the World Health Organization, cardiovascular disease (CVD) remains the leading cause of death globally, responsible for an estimated 17.7 million deaths in 2015^[1]^. Among these, heart failure (HF) has emerged as a global pandemic, with an estimated 64.3 million people suffering from HF worldwide as of 2017^[2]^. HF is a clinical syndrome traditionally defined as the heart’s reduced ability to pump or fill with blood or as an abnormality in cardiac structure or function that results in inadequate cardiac output. This can also involve compensatory neurohormonal activation and increased left ventricular filling pressure^[2]^.

HF is commonly classified into three types based on left ventricular ejection fraction (LVEF): heart failure with reduced ejection fraction (HFrEF, LVEF <40%), mid-range ejection fraction (LVEF 40%–49%), and preserved ejection fraction (HFpEF, LVEF ≥50%)^[3]^. However, this LVEF-based classification has been criticized for oversimplifying a complex condition. While HF prevalence continues to rise, the HFpEF phenotype is becoming more common, primarily due to advancements in diagnostic tools, increased clinical awareness, and higher life expectancy^[4]^. Currently, HFpEF accounts for nearly half of all HF cases in the United States, with around 2.5 million diagnoses. Despite its growing prevalence, the pathophysiology of HFpEF remains poorly understood^[5]^. One key factor in the development of HF is restricted blood flow to tissues and organs caused by constrictions in both the larger coronary vessels and the microvasculature, which play crucial roles in determining patient outcomes^[6]^. Research has shown that disruptions in microcirculation contribute to varying degrees of HF severity, with the extent of microvascular dysfunction (MVD) significantly influencing the disease’s progression and overall pathophysiology.

Traditionally, HFpEF was attributed to myocardial stiffness caused by long-term hypertension and increased circulatory demand, leading to concentric hypertrophy and diastolic dysfunction^[5]^. However, age is not the only factor contributing to the rising incidence of HF. Comorbidities such as obesity and type 2 diabetes (T2D) also play significant roles. Worryingly, younger populations—including children, adolescents, and adults under 30—are at increased risk of HF due to the rising prevalence of T2D and obesity^[7]^. T2D, the most common metabolic disease, has reached pandemic proportions, affecting approximately 382 million people in 2013, with projections suggesting this number will rise to 590 million by 2035^[8]^.

MVD plays a pivotal role in the progression of HF by impairing coronary microcirculation, which leads to inadequate myocardial perfusion^[9]^. Current therapeutic strategies focus on improving blood flow and microvascular function. Pharmacological agents, such as beta-blockers, angiotensin II receptor blockers (ARBs), and angiotensin-converting enzyme (ACE) inhibitors, are frequently used to reduce small vessel resistance^[9,10]^. New drugs like soluble guanylate cyclase stimulators and sodium-glucose cotransporter-2 (SGLT2) inhibitors have shown promise in improving microvascular function. Lifestyle modifications, including regular physical activity, are also recommended to enhance endothelial function and reduce the burden of HF^[9–11]^.

MVD exacerbates HF outcomes by reducing blood supply to the heart, leading to diminished cardiac performance and increased morbidity and mortality. While traditional medications, such as ACE inhibitors and beta-blockers, provide symptom relief, newer therapies such as SGLT2 inhibitors and statins can potentially improve vascular health and clinical outcomes^[12]^.

In advanced cases of HF, modern therapeutic approaches emphasize promoting endothelial function, reducing inflammation, and managing cardiac risk factors through pharmacological and lifestyle interventions. For patients unresponsive to medical management, surgical options such as coronary artery bypass grafting or transmyocardial laser revascularization may be considered. In extreme cases, techniques like ventricular assist devices or heart transplantation may be used to support circulation and improve prognosis^[13]^.

## Physiology of microvascular function

### Normal microvascular function

Microcirculation, essential for gas and nutrient exchange, comprises capillaries, arterioles, and venules^[12]^. Various mechanisms regulate blood flow. Autoregulation adjusts arterial diameter based on local tissue needs, while hormones and the autonomic nervous system control blood flow at a broader level^[14,15]^.

### Anatomy and physiology of the microcirculation

The coronary vessels include epicardial arteries (>400 µm), pre-arterioles (100–400 µm), arterioles (<100 µm), and capillaries (<10 µm). The epicardial arteries serve as capacitance conduits, responding to shear stress and enabling dilation via the endothelium. Although they comprise only 5%–10% of coronary vascularization, these structures are sometimes visible during coronary angiography^[16]^. Vascular resistance, influenced by blood viscosity and vessel diameter, regulates blood flow. Shortening of vessels increases resistance, reducing blood flow. Smooth muscle tone and endothelial factors like nitric oxide (NO) significantly impact this process^[14,15]^. Regular microvascular activity involves precise blood flow regulation through microcirculatory systems, including small arteries, arterioles, capillaries, and venules^[17]^.

### Regulation of blood flow and vascular resistance

The endothelium is vital for maintaining microvascular function, releasing substances that regulate vasodilation and vasoconstriction. Under normal conditions, a balance between vasodilators (e.g., NO) and vasoconstrictors ensures adequate blood flow, oxygen delivery, and nutrient exchange^[18]^. Microcirculation adapts dynamically to fluctuations in tissue metabolic demands. During increased physical activity, blood flow to active tissues rises due to adjustments in microvascular function. Shear stress also triggers endothelial cells (EC) to release NO, inducing vasodilation and enhancing perfusion. These mechanisms underscore the importance of microvascular health in maintaining cardiovascular stability^[18,19]^.

### Pathophysiology of MVD in HF

In HF, coronary microvascular dysfunction (CMD) arises from both endothelium-dependent and endothelium-independent pathways. Endothelium-dependent dysfunction occurs when there is an imbalance between the relaxing agent NO and the constrictor endothelin-1, a common finding in patients with HFpEF. These individuals typically have higher concentrations of endothelin-1 and lower levels of NO^[19]^. Endothelium-independent dysfunction results from mismatched vasoconstrictors and vasodilators, leading to altered vascular tone. Endothelium-dependent dysfunction is observed in 29% of HFpEF patients, while endothelium-independent dysfunction is found in 33% of patients, associated with worsening diastolic function and increased mortality^[20,21]^.

### Mechanisms contributing to MVD in HF

Chronic low-grade inflammation and oxidative stress are prominent contributors to MVD in HF. Conditions such as obesity, T2D mellitus, hypercholesterolemia, and hypertension—collectively known as metabolic syndrome (MetS)—cause direct damage to the coronary endothelium and promote chronic low-grade inflammation throughout the body^[22]^.

### Endothelial dysfunction and its role

Immunocytes move through the activated EC, responsible for developing low-grade chronic heart bloody tissue inflammation. In addition, oxidative stress occurs in endothelium and cardiomyocytes (CM) when there is an imbalance between the production of reactive oxygen species (ROS) and antioxidant defenses. The formation of ROS is further increased by immune mediators such as interferon-γ, tumor necrosis factor (TNF)-α, and interleukin 1 (IL)-1β. Extended ROS-mediated activation of the inflammasome and elevated levels of transforming growth factor-β modify the expression of genes associated with fibrosis, ultimately leading to cardiac fibrosis^[23]^.

Extreme oxidative stress can lead to alterations of DNA, lipids, and proteins. This leads to malfunctioning mitochondria, impairing the capacity of CM to produce ATP, manage calcium, and contract. In addition, ROS-induced protein alterations such as S-nitrosylation lead to a decrease in endothelial nitric oxide synthase (eNOS)-mediated NO generation and sarcomeric myofilament dysfunction^[24]^. Moreover, excessive levels of ROS lead to an uncoupling of the eNOS enzyme that disrupts both the myocardial perfusion and flow-mediated vasodilation. Thus, it worsens the energy supply-demand balance in CM. In addition, this is additionally reflected in heightened levels of nitrosative stress due to increased cardiac activation of inducible nitric oxide synthase. Finally, prolonged vascular pro-inflammatory activation and oxidative stress may induce death or apoptosis among EC, reducing blood flow into the heart and causing blood vessel loss or disappearance^[25]^.

## Clinical impact of MVD

### Diagnostic approaches

Assessing CMD requires a comprehensive approach that combines advanced imaging techniques with biomarker analysis. This integrated method provides essential insights into the health and performance of the microvasculature, leading to more accurate diagnoses, better risk assessment, and personalized treatment plans^[26–28]^.

### Imaging techniques

Various imaging methods are used to assess microvascular function, offering unique perspectives on different aspects of CMD. When used together, they provide a complete view of the disease.

#### Positron emission tomography (PET)

PET is the gold standard for evaluating myocardial blood flow and coronary flow reserve (CFR). Using radiotracers, PET measures blood perfusion through coronary arteries and the myocardium, detecting microvascular abnormalities that may be missed with other imaging techniques. PET’s ability to quantify the heart’s capacity to increase blood flow under stress makes it particularly useful in diagnosing CMD^[29]^. This insight into the heart’s blood flow dynamics is crucial for understanding the broader impact of CMD^[30,31]^.

#### Cardiac magnetic resonance (CMR) imaging

CMR provides a detailed look at myocardial perfusion and microvascular resistance, evaluating the myocardial perfusion reserve (MPR)—the blood flow ratio during stress versus at rest. This technique offers valuable insights into the integrity of the microvasculature^[32,33]^. In addition, CMR can measure the index of microcirculatory resistance, further enriching our understanding of microvascular health^[34]^. This makes CMR an essential tool in diagnosing and managing CMD.

#### Dynamic myocardial perfusion computed tomography (CT)

Dynamic myocardial perfusion CT uses iodine contrast to assess both the myocardium and coronary arteries, providing high-resolution images that evaluate both global and focal blood supply to the myocardium. This makes it a powerful tool for identifying CMD^[35]^. The ability to capture detailed images of the heart’s anatomy and blood flow enhances its utility in CMD detection^[36,37]^.

#### Transthoracic Doppler echocardiography (TTDE)

TTDE is a noninvasive method of measuring CFR by assessing blood flow velocity in the coronary arteries. It evaluates the ability of the coronary arteries to dilate in response to increased blood flow demand, making it a valuable tool for detecting CMD^[38]^. TTDE is more accessible and cost-effective than PET or CMR, making it a practical option for many healthcare settings^[39]^. While it may not offer the same level of detail, it remains a valuable tool, especially in environments with limited resources.

### Biomarkers

Circulating biomarkers are crucial in noninvasive CMD evaluation. These biomarkers reflect pathological processes such as inflammation, oxidative stress, endothelial dysfunction, and coagulation, providing insight into the underlying disease mechanisms^[40]^.

#### Inflammatory biomarkers

Markers like C-reactive protein (CRP) and high-sensitivity CRP (hs-CRP) are often elevated in patients with CMD. These markers are acute-phase reactants produced in response to inflammatory cytokines such as interleukin-6 (IL-6), and their presence is associated with an increased risk of MVD^[41,42]^. Elevated levels of CRP and hs-CRP indicate chronic inflammation, which can impair endothelial function and contribute to the progression of CMD^[43,44]^.

#### Oxidative stress biomarkers

Superoxide dismutase and paraoxonase are critical indicators of oxidative stress, reflecting the balance between ROS and antioxidant defenses. In CMD, an increase in ROS results in oxidative damage to EC, reducing NO availability and leading to vascular dysfunction. Elevated levels of superoxide free radicals and reduced paraoxonase activity are markers of oxidative stress and are associated with worse clinical outcomes in CMD patients^[45–47]^.

#### Endothelial dysfunction biomarkers

Asymmetric dimethylarginine (ADMA) and endothelin-1 are crucial biomarkers of endothelial health. ADMA inhibits NO synthase, thereby reducing NO production, while endothelin-1 acts as a potent vasoconstrictor produced by EC^[48]^. Elevated levels of these biomarkers are closely associated with impaired endothelial function and increased vascular resistance, both prominent in CMD^[49]^.

#### Coagulation biomarkers

Von Willebrand factor and plasminogen activator inhibitor-1 are typically elevated in CMD, signifying a prothrombotic state. These markers contribute to an increased risk of thrombotic events in the microvasculature, which can further complicate the condition^[50]^. Monitoring these biomarkers can help in assessing the thrombotic risk associated with CMD.

By combining advanced imaging techniques with the analysis of circulating biomarkers, clinicians can achieve a more comprehensive understanding of MVD. Imaging directly assesses microvascular function, while biomarkers offer valuable molecular insights into the underlying pathological processes. This integrative approach enables more accurate diagnoses, better risk stratification, and more tailored therapeutic interventions, ultimately improving clinical outcomes for patients with CMD.

### Correlation between the severity of MVD (CMD) and the stages of HF

There is a well-documented relationship between the severity of CMD and the stages of HF, particularly in heart failure with HFpEF and, to a lesser extent, in HFrEF^[51]^.

#### CMD in HFpEF

CMD is highly prevalent among patients with HFpEF. Studies, such as the PROMIS-HFpEF trial, have shown that up to 75% of patients with HFpEF exhibit CMD. Those with CMD experience higher rates of cardiovascular death and hospitalization due to HF^[51,52]^. The severity of CMD, often defined by reduced CFR, correlates with worse clinical outcomes. CMD in HFpEF also leads to impaired left ventricular diastolic function, exacerbating the condition by increasing myocardial ischemia and worsening diastolic dysfunction^[53]^.

#### CMD in HFrEF

Although less explored in HFrEF compared to HFpEF, CMD still plays a significant role. CMD in HFrEF is often associated with decreased CFR, higher left ventricular end-diastolic pressure, and reduced myocardial contractile reserve. These impairments contribute to the progression of HF and higher mortality rates. Studies have shown that lower CFR in HFrEF patients is linked to an increased risk of major adverse cardiovascular events, including the progression of HF^[53,54]^.

#### CMD severity and HF stages

The severity of CMD is correlated with the progression of HF in both HFpEF and HFrEF. In HFpEF, CMD primarily affects diastolic function, leading to more severe symptoms and worse clinical outcomes^[55]^. In HFrEF, CMD compromises myocardial contractile reserve and overall cardiac function, resulting in poorer clinical outcomes^[56]^. In addition, CMD is strongly associated with an increased risk of major adverse cardiovascular events (MACE) and all-cause mortality, particularly in patients with nonobstructive coronary artery diseases^[57]^.

Recent studies also suggest that CMD may serve as a predictor for cancer incidence, with lower cancer-free survival rates observed in CMD patients compared to those without CMD across various demographics, including age, sex, and common comorbidities such as hypertension and diabetes^[58,59]^. Furthermore, CMD’s predictive value extends to patients with a history of cancer, where it is linked to a higher incidence of cardiovascular events, underscoring its role in patient risk stratification and management^[58]^.

## Mechanisms linking MVD and HF

### Inflammatory pathways and the role of inflammation in MVD

Inflammation is a critical driver in the pathogenesis of CMD and is strongly linked to the development of HF^[60]^. CMD often results from structural and functional abnormalities in the coronary microvessels, primarily caused by inflammatory processes targeting the EC lining these vessels^[61]^. Inflammation plays a pivotal role in bridging MVD and HF by promoting endothelial dysfunction, impaired coronary blood flow, and myocardial ischemia.

### Inflammatory pathways in CMD

The inflammatory response in CMD is initiated by activating cell adhesion molecules, leading to the infiltration of inflammatory cells. These leukocytes adhere to EC through adhesion molecules such as P-selectin, ICAM-1, and VCAM-1, which are upregulated in response to vascular injury or stress^[62–64]^. The infiltration of these immune cells triggers the release of inflammatory mediators such as cytokines (TNF-α, IL-1β, IL-6), further exacerbating endothelial dysfunction^[62,65,66]^. This inflammatory cascade perpetuates ongoing vascular damage, leading to impaired coronary flow and the progression of HF^[66]^.

### Role of inflammation in CMD and HF

Inflammation-induced CMD is especially detrimental in the context of HF. In HFpEF, CMD is closely linked to systemic inflammation, and elevated inflammatory markers such as CRP are commonly observed^[67,68]^. These markers are correlated with reduced CFR, a hallmark of CMD, and are associated with worse clinical outcomes in HFpEF patients^[69,70]^. Persistent inflammation impairs myocardial relaxation, increases myocardial stiffness, and aggravates HF symptoms^[71,72]^.

Although CMD is less studied in HFrEF, it still worsens myocardial function. Inflammatory CMD in HFrEF leads to reduced myocardial contractile reserve and increased left ventricular end-diastolic pressure, accelerating HF progression^[54,73]^. Targeting inflammation through therapeutic interventions aimed at cytokine modulation or leukocyte adhesion inhibition may help mitigate CMD and improve clinical outcomes in HF patients^[74,75]^.

### Impact of CMD on cardiac remodeling and function

CMD significantly contributes to both structural and functional alterations in the heart, playing a central role in the progression of HF. CMD mainly influences the structural remodeling of the left ventricle (LV) alongside functional changes, exacerbating HF through various mechanisms^[76]^.

#### Left ventricular remodeling

CMD is closely associated with adverse left ventricular remodeling, particularly following myocardial infarction (such as ST-segment elevation myocardial infarction [STEMI]). Despite successful revascularization, CMD can persist, leading to inadequate myocardial perfusion and ischemia^[77,78]^. This ongoing ischemia triggers a cascade of pathological events, including CM death, chronic inflammation, and fibrosis. These processes result in adverse remodeling, which is characterized by an increase in left ventricular end-diastolic volume and a reduction in ejection fraction (EF), leading to a stiffer and less compliant LV^[79,80]^.

#### Diastolic dysfunction

CMD also plays a critical role in the progression of diastolic dysfunction. This condition is marked by impaired relaxation and increased LV stiffness, leading to elevated filling pressures and the typical symptoms of HF^[81]^. CMD exacerbates diastolic dysfunction by promoting myocardial fibrosis and reducing CFR, thus increasing myocardial ischemic burden. Over time, patients with CMD often progress from grade 1 to grade 2 diastolic dysfunction, which correlates with poorer clinical outcomes, including higher rates of hospitalization and increased mortality^[82,83]^.

CMD’s significant impact on cardiac remodeling and function underscores the importance of early diagnosis and management, particularly in post-myocardial infarction patients. CMD is a strong predictor of poor clinical outcomes, such as major adverse cardiac events (MACE), HF progression, and mortality^[57,77]^. Therapeutic interventions targeting CMD by improving microvascular function and reducing ischemia may help prevent adverse remodeling and enhance patient outcomes.

### Effects of metabolic disorders on microvascular function

Metabolic disorders, such as diabetes and MetS, significantly impact microvascular function, particularly CMD^[84,85]^. These conditions lead to a range of vascular abnormalities that heighten the risk of CVD by exacerbating MVD.

#### Diabetes and MVD

T2D is a primary contributor to MVD, with hyperglycemia playing a central role. Persistent high blood glucose levels lead to endothelial dysfunction, a critical factor in the development of CMD^[86]^. Hyperglycemia causes the glycation of proteins and lipids, forming advanced glycation end products (AGEs)^[87,88]^. These AGEs bind to receptors on EC, initiating inflammatory pathways that exacerbate endothelial damage and reduce NO availability^[89]^. The reduction of NO impairs vasodilation, a hallmark of endothelial dysfunction, leading to increased vascular resistance and compromised microcirculatory blood flow^[90]^.

In addition, insulin resistance, frequently associated with T2D, further contributes to CMD. Insulin resistance leads to elevated levels of free fatty acids, which promote oxidative stress and additional endothelial damage^[91]^. This oxidative stress activates pro-inflammatory cytokines, exacerbating vascular inflammation and reducing NO bioavailability, thereby perpetuating MVD^[92]^.

#### MetS and MVD

MetS, which encompasses conditions like obesity, hypertension, dyslipidemia, and insulin resistance, also plays a significant role in worsening MVD. Each component of MetS contributes to vascular abnormalities that aggravate CMD^[93–95]^.

Obesity, a core aspect of MetS, is linked to increased adiposity, which raises adipokines and inflammatory cytokines such as TNF-α and IL-6^[96]^. These inflammatory factors impair endothelial function by reducing NO production and inducing oxidative stress, further contributing to MVD^[97]^.

Moreover, activation of the renin-angiotensin-aldosterone system (RAAS) in MetS worsens MVD by promoting vasoconstriction and sodium retention, which elevate blood pressure and increase the risk of vascular injury^[98]^. Altered lipid metabolism, characterized by elevated triglycerides and low HDL cholesterol, leads to lipotoxicity, which damages EC^[99]^. Hyperinsulinemia, another component of MetS, further compounds this endothelial damage. It promotes vascular smooth muscle cell proliferation and fibrosis, aggravating CMD^[100,101]^.

## Therapeutic strategies targeting HF and MVD

HF is a complex clinical condition characterized by the heart’s inability to pump blood effectively, resulting in insufficient blood flow and fluid congestion in the lungs and tissues^[56]^. HF can stem from various causes, including ischemic heart disease, valvular heart disease, systemic hypertension, or MVD. Compromised heart function leads to symptoms such as fatigue, dyspnea, and edema, all of which reduce a patient’s quality of life (QoL) and functional capacity^[102]^. Given the complexity of HF, a multifaceted treatment approach is necessary to address symptoms, improve QoL, and reduce mortality.

Early intervention with lifestyle modifications is crucial in managing HF. Dietary changes, regular physical activity, and weight management are essential components of any HF treatment plan^[103]^. These interventions help reduce the strain on the heart and can slow disease progression, making them a foundational part of therapeutic strategies.

### Pharmacological advances in managing HF

Several pharmacological treatments have emerged in recent years, significantly improving patient outcomes.

#### Angiotensin receptor-neprilysin inhibitors (ARNIs)

One of the notable recent developments is the introduction of ARNIs, such as sacubitril/valsartan. These agents combine vasodilatory and natriuretic properties, helping to reduce hospitalizations and improve the QoL in HF patients^[104]^. ARNIs represent a significant advancement in HF management by addressing multiple pathways involved in disease progression.

#### Sodium-glucose cotransporter 2 (SGLT2) inhibitors

Another significant advance is using SGLT2 inhibitors, such as dapagliflozin and empagliflozin. Initially developed for glycemic control in diabetic patients, these drugs have shown remarkable benefits in HF, independent of their glucose-lowering effects. They help reduce HF symptoms and improve survival rates, representing a paradigm shift in HF treatment^[105]^.

#### Diuretics

Loop diuretics remain one of the most traditional treatment options for HF, particularly for the relief of venous congestion. These medications help reduce fluid retention, alleviating symptoms like peripheral edema. However, distinguishing between cardiogenic and non-cardiogenic edema can be challenging, complicating the precise use of diuretics in some cases^[106]^. Research has also highlighted the relationship between serum bicarbonate levels and HF, further underscoring the importance of diuretics in managing this condition^[107]^.

A study by Imaeda et al. compared the efficacy of long-acting diuretics with short-acting loop diuretics following hospitalization due to HF. Their findings showed that long-acting diuretics were associated with better outcomes than their short-acting counterparts, offering insights into improving the management of HF symptoms post-hospitalization^[108]^.

While significant advancements have been made in HF treatment, diuretics remain essential for managing congestion in symptomatic patients. Loop diuretics continue to be the first line of treatment due to their efficacy in relieving fluid buildup^[109]^. As the field progresses, there is an increasing recognition of the need for innovative approaches to prevent and restore heart function. Maintaining appropriate diuretic doses, choosing diuretic agents, and addressing diuretic resistance are all critical considerations in managing HF patients on long-term diuretic therapy.

#### Nesiritide

Nesiritide, a recombinant form of human B-type natriuretic peptide, has positively affected HF management by improving hemodynamic parameters and patient symptoms. In a study conducted by Zhao et al., nesiritide showed superior outcomes in increasing LVEF, cardiac index, and urine output while simultaneously reducing pulmonary capillary wedge pressure and natriuretic peptide levels in patients with HF and acute myocardial infarction. Despite these benefits, nesiritide did not impact mortality, heart rate, readmission rates for hypotension, or renal dysfunction compared to other treatments. The study concluded that nesiritide is safe and significantly improves this patient group’s cardiovascular health and well-being^[110]^.

### Therapeutic strategies for CMD

MVD, characterized by impaired blood flow in small vessels, is a growing focus in cardiovascular research due to its role in conditions such as microvascular angina (MVA) and CMD^[111]^. This dysfunction reduces CFR and endothelial dysfunction, impairing myocardial perfusion and ischemia^[102]^. CMD is frequently under-recognized in clinical practice, creating a need for enhanced diagnostic and therapeutic approaches. Managing CMD remains challenging due to the condition’s diverse presentation and varying diagnostic methods.

#### Beta-blockers

Managing CMD involves risk factor management, pharmacological interventions, and patient-centered strategies. Beta-blockers are commonly prescribed to alleviate symptoms of effort angina by lowering myocardial oxygen demand. Third-generation beta-blockers, such as nebivolol and carvedilol, offer additional benefits through NO-mediated vasodilation, reducing oxygen requirements by lowering inotropy, heart rate, and blood pressure. This promotes longer diastolic time and enhances coronary perfusion. These medications have decreased the frequency of anginal episodes, improved exercise capacity, and enhanced endothelial function, thus improving coronary artery blood flow^[112]^.

#### ACE inhibitors and ARBs

Angiotensin-converting enzyme inhibitors (ACEIs) and angiotensin receptor blockers (ARBs) have been extensively studied for their role in improving microvascular circulation. ACEIs, such as enalapril and quinapril, enhance exercise performance and CFR by reducing oxidative stress and increasing NO synthesis^[113,114]^. For instance, Pauly et al. demonstrated that after 16 weeks of quinapril therapy, patients experienced a significant increase in CFR and a reduction in anginal symptoms compared to placebo^[115]^. Although ARBs, such as irbesartan, have shown some benefits in improving CFR, the evidence supporting their efficacy could be stronger for ACEIs. Baric et al. reported that in hypertensive patients, coronary flow velocity reserve significantly improved after three months of irbesartan treatment, alongside a notable reduction in blood pressure^[116]^.

#### NO modulators

NO modulators, including nitrates, sildenafil, L-arginine, tetrahydrobiopterin (BH4), and cilostazol, have shown varying degrees of efficacy in managing CMD. Nitrates are well-known for decreasing myocardial oxygen demand but can sometimes impair endothelial function. Cilostazol and calcium channel blockers (CCBs) have demonstrated potential in alleviating vasospastic angina^[117]^. BH4, which promotes NO-mediated vasodilation, holds promise but requires further clinical validation. A study by Russo et al. investigated the effects of short-acting nitrates on exercise stress tests (EST) in patients with MVA and obstructive coronary artery disease (CAD). The findings indicated that short-acting nitrates improved EST results in patients with obstructive CAD but had limited efficacy in MVA, possibly due to a diminished ability to dilate the microvasculature further^[118]^.

#### Calcium channel blockers

CCBs have been demonstrated to reduce microvascular tone and spasm, potentially improving CFR and easing symptoms in CMD. CCBs are particularly useful in treating angina pectoris due to small-vessel CAD and impaired vasodilator response. Research by Cannon et al. revealed that both verapamil and nifedipine significantly reduced angina frequency, lowered the need for nitroglycerin, and improved exercise duration compared to placebo. These benefits underscore the role of CCBs in managing angina related to CMD and small-vessel CAD^[119,120]^.

### Emerging therapies for CMD

Emerging therapeutic options for CMD include nonsteroidal anti-inflammatory drugs such as colchicine and interleukin-1 (IL-1) blockers, which may prevent endothelial inflammation and reduce the risk of HF and stroke. IL-1 inhibitors have been shown to significantly lower the risk of HF, while colchicine has been linked to a reduction in stroke incidence^[121]^. Statins have also been found to improve CFR and endothelial function, with clinical trials showing enhanced exercise tolerance and improved CFR in patients with MVA^[122,123]^. SGLT2 inhibitors, such as empagliflozin, have shown promise in enhancing CFR, particularly in diabetic models. In a study by Tu et al., empagliflozin improved coronary microvascular function in diabetic mice by reducing cardiac fibrosis, preserving cardiac pericytes, and enhancing CFR, indicating potential cardiovascular benefits in diabetic heart disease^[124]^. Antioxidants such as vitamins C and E are still being researched for their potential to counteract oxidative stress and maintain microvascular integrity. Metabolic modulators like trimetazidine and ranolazine are also being explored as treatments for microvascular diseases linked to metabolic dysfunctions. In a pilot randomized trial, patients receiving ranolazine showed improved scores on the Seattle Angina Questionnaire, with a trend toward improved myocardial perfusion rates compared to placebo^[125]^. These therapies contribute to a holistic CMD management model, focusing on symptomatic relief and long-term cardiovascular health.

## Impact of MVD on specific HF phenotypes

MVD plays a pivotal role in the pathophysiology of HF with HFrEF and HFpEF. CMD has emerged as a critical component in HFpEF as research has progressed. CMD contributes significantly to HFpEF, often characterized by reduced CFR and MPR. Histological studies frequently demonstrate coronary microvascular rarefaction in HFpEF patients, which correlates with worse clinical outcomes, such as higher hospitalization rates and major adverse cardiovascular events (MACE)^[111,126]^. Concurrently, studies on coronary endothelial dysfunction (CED) underscore its considerable impact on the risk of developing atrial fibrillation (AF). Corban et al. found that among patients presenting with chest pain but without obstructive CAD, CED strongly predicted a higher incidence of AF over a 10.5-year follow-up. Specifically, 12% of these patients developed AF, with 97% of the cases occurring in those with preexisting CED. The presence of CED was linked to an 11% higher absolute risk and a 5.8-fold increase in relative risk of AF compared to patients with normal endothelial function. These findings highlight the importance of maintaining coronary endothelial health to prevent AF, particularly in patients without obstructive CAD^[127]^.

MVD remains a crucial contributor to HFrEF. Research indicates that impaired peripheral microvascular endothelial function closely correlates with outcomes in HFrEF patients. This link is attributed to increased oxidative stress, reduced endothelium-derived NO production, and RAAS activation. These mechanisms drive the decline in microvascular function, emphasizing the need for targeted treatments to enhance HFrEF patient outcomes^[128]^. A study by Tromp et al. examined microvascular disease in patients with diabetes and HF, comparing HFrEF and HFpEF groups. The study included 601 diabetic participants, of whom 21.5% had microvascular complications. Diabetic patients with any microvascular complications were more likely to have HFpEF, with an odds ratio of 1.70 (95% CI: 1.15–2.50; *P* = 0.008). Furthermore, the likelihood of HFpEF increased with the number of microvascular complications. The study also found that microvascular complications were associated with greater left ventricular (LV) hypertrophy and a more pronounced reduction in QoL in HFpEF patients compared to HFrEF patients^[129]^.

In addition, the PROMIS-HFpEF study showed that reduced CFR correlates with markers of HF severity and systemic endothelial dysfunction. This highlights a strong connection between CMD and the inflammatory processes observed in HFpEF. These insights emphasize the significant role of microvascular complications in HFpEF outcomes and underscore the need for targeted therapies addressing both MVD and HF severity^[51]^.

## Future directions and research opportunities

MVD in HF represents a complex challenge in both clinical practice and research. A significant gap remains in our understanding of the mechanisms underlying MVD and its impact on HF progression. Despite advancements, much is still unknown about the intricate processes involved and how they contribute to HF. The variability of MVD across different HF subtypes, such as HFpEF and HFrEF, further complicates this issue. Translating research insights into clinical practice presents substantial challenges due to the complexity of the mechanisms involved, the variability in patient responses, and the absence of standardized diagnostic tools. In addition, the need for specialized expertise and resources to assess and manage MVD in HF adds logistical difficulties in clinical settings.

Promising research areas in MVD offer potential breakthroughs in diagnostics and therapies.

Advances in imaging techniques, such as PET and cardiac magnetic resonance imaging (MRI), provide new opportunities to visualize and measure microvascular function in HF patients. Furthermore, innovative therapeutic approaches targeting MVD, including endothelial-targeted therapies and microvascular vasodilators, show potential for improving outcomes in patients with microvascular abnormalities. The role of personalized medicine in addressing MVD in HF is crucial. Tailoring treatment strategies to individual patient characteristics such as genetic predispositions, comorbidities, and specific microvascular function aspects can enhance therapeutic effectiveness and patient care. Personalized medicine aims to identify and target each patient’s unique mechanisms of MVD, leading to more effective and customized treatment plans.

Future research should focus on bridging gaps in understanding, addressing challenges in translating research findings into clinical practice, and exploring new diagnostic and therapeutic strategies. By enhancing knowledge of MVD mechanisms and implementing personalized treatment approaches, researchers can improve outcomes and QoL for HF patients with microvascular abnormalities.

## Conclusion

MVD is a crucial driver of HF, significantly impacting both HF with HFpEF and HFrEF. As the understanding of CMD expands, its role in worsening cardiac function, exacerbating comorbidities, and contributing to adverse outcomes such as major cardiovascular events and AF becomes increasingly apparent. Targeting microvascular health through traditional and emerging therapeutic strategies offers hope for improving HF management, particularly in addressing the unique challenges posed by HFpEF, where CMD plays a more pronounced role.

The future of HF treatment lies in integrating advanced diagnostic tools, such as PET and CMR, combined with developing personalized therapeutic strategies. Innovations in pharmacological interventions, including SGLT2 inhibitors and endothelial-targeted therapies, can potentially enhance microvascular function and improve patient outcomes. As research progresses, a deeper understanding of the molecular and inflammatory pathways linking MVD to HF could revolutionize the prevention and management of this complex condition. Addressing MVD in HF offers a pathway to improve clinical outcomes and reduce the global burden of HF, thus marking a crucial frontier in cardiovascular research and therapeutic development.

## Data Availability

Not applicable.
